# Oral chondroitin sulfate and prebiotics for the treatment of canine Inflammatory Bowel Disease: a randomized, controlled clinical trial

**DOI:** 10.1186/s12917-016-0676-x

**Published:** 2016-03-10

**Authors:** Sergi Segarra, Silvia Martínez-Subiela, Marta Cerdà-Cuéllar, Daniel Martínez-Puig, Alberto Muñoz-Prieto, Fernando Rodríguez-Franco, Antonio Rodríguez-Bertos, Karin Allenspach, Alfonso Velasco, José Cerón

**Affiliations:** R&D Bioiberica SA, Pça. Francesc Macià 7, 08029 Barcelona, Spain; Interlab-UMU, Campus de Excelencia “Mare Nostrum”, University of Murcia, Campus Espinardo, 30071 Murcia, Spain; Centre de Recerca en Sanitat Animal (CReSA), IRTA, Campus de la Universitat Autònoma de Barcelona, 08193 Bellaterra, Cerdanyola del Vallès Spain; Department of Animal Medicine and Surgery, Faculty of Veterinary Science, Complutense University of Madrid (UCM), 28040 Madrid, Spain; Department of Animal Medicine and Surgery, Faculty of Veterinary Science, and Health Surveillance Centre (VISAVET), Complutense University of Madrid (UCM), 28040 Madrid, Spain; Department of Veterinary Clinical Sciences and Services, Royal Veterinary College, University of London, Hatfield, Hertfordshire EN6 1NB UK

**Keywords:** Chronic enteropathy, Dog, Glycosaminoglycan, Resistant starch, β-glucans, Oxidative stress, CIBDAI

## Abstract

**Background:**

Canine inflammatory bowel disease (IBD) is a chronic enteropathy of unknown etiology, although microbiome dysbiosis, genetic susceptibility, and dietary and/or environmental factors are hypothesized to be involved in its pathogenesis. Since some of the current therapies are associated with severe side effects, novel therapeutic modalities are needed. A new oral supplement for long-term management of canine IBD containing chondroitin sulfate (CS) and prebiotics (resistant starch, β-glucans and mannaoligosaccharides) was developed to target intestinal inflammation and oxidative stress, and restore normobiosis, without exhibiting any side effects. This double-blinded, randomized, placebo-controlled trial in dogs with IBD aims to evaluate the effects of 180 days administration of this supplement together with a hydrolyzed diet on clinical signs, intestinal histology, gut microbiota, and serum biomarkers of inflammation and oxidative stress.

**Results:**

Twenty-seven client-owned biopsy-confirmed IBD dogs were included in the study, switched to the same hydrolyzed diet and classified into one of two groups: supplement and placebo. Initially, there were no significant differences between groups (*p* > 0.05) for any of the studied parameters. Final data analysis (supplement: *n* = 9; placebo: *n* = 10) showed a significant decrease in canine IBD activity index (CIBDAI) score in both groups after treatment (*p* < 0.001). After treatment, a significant decrease (1.53-fold; *p* < 0.01) in histologic score was seen only in the supplement group. When groups were compared, the supplement group showed significantly higher serum cholesterol (*p* < 0.05) and paraoxonase-1 (PON1) levels after 60 days of treatment (*p* < 0.01), and the placebo group showed significantly reduced serum total antioxidant capacity (TAC) levels after 120 days (*p* < 0.05). No significant differences were found between groups at any time point for CIBDAI, WSAVA histologic score and fecal microbiota evaluated by PCR-restriction fragment length polymorphism (PCR-RFLP). No side effects were reported in any group.

**Conclusions:**

The combined administration of the supplement with hydrolyzed diet over 180 days was safe and induced improvements in selected serum biomarkers, possibly suggesting a reduction in disease activity. This study was likely underpowered, therefore larger studies are warranted in order to demonstrate a supplemental effect to dietary treatment of this supplement on intestinal histology and CIBDAI.

## Background

Inflammatory bowel disease (IBD) in dogs is a chronic gastrointestinal tract disorder of unknown cause [1, 2], although the mucosal immune system, genetic susceptibility, and the enteric microenvironment (nutrition and microbiota) are considered to be important in its pathogenesis [3–5]. Treatment protocols for IBD combine diet modification, antibacterials and immunosuppressive therapy [6], aiming at reducing intestinal inflammation and restoring gut normobiosis. Dietary modification is considered an adequate therapeutic starting point, since most IBD dogs respond positively to dietary management alone [7]. Dogs that do not respond or respond partially, require further treatment with antibiotics and/or immunosuppressive drugs like glucocorticoids, which can be associated with severe side effects [4, 8–10]. Ideally, any long-term treatment protocol for IBD should aim at maintaining patients in clinical remission for as long as possible by adequately combining such therapeutic modalities while intending to minimize associated side effects.

Chondroitin sulfate (CS), a natural glycosaminoglycan present in the extracellular matrix, has been suggested to reduce the incidence and severity of relapses in human IBD [11]. CS inhibits nuclear factor kappa-light-chain-enhancer of activated B cells (NF-κB) activity [11], which is also a suggested component of the anti-inflammatory activity of glucocorticoids and cyclosporine A [12]. NF-κB is markedly increased in IBD [11, 13]. Oral CS might therefore reduce intestinal inflammation and benefit dogs with IBD.

Oxidative stress is also believed to play a key role in the pathogenesis of IBD [14]. Paraoxonase-1 (PON1), an antioxidant enzyme used as a biomarker of oxidative stress and inflammation in human IBD [15], could also serve as a biomarker in dogs with IBD given the similarities between human and canine IBD. In human IBD, lower PON1 serum concentrations are associated with increased inflammation and disease activity [16]. Decreasing oxidative stress, shown as an increased PON1, could therefore be an effective endpoint for IBD treatment. Decreased serum TAC levels have been reported in human patients with ulcerative colitis and Crohn’s disease [17].

Lower bacterial diversity and altered microbial communities have been reported in canine IBD [2, 4, 5, 18, 19]. Orally administered prebiotics promote the growth of beneficial gut microbiota [20–22]. Short chain fatty acids (SCFA), especially butyrate, which is the preferential source of energy for colonocytes, help maintaining the intestinal mucosal barrier [23, 24]. A reduction in SCFA producing bacteria, especially *Faecalibacterium* spp. and Fusobacteria, has been reported in dogs with IBD [5, 19]. Large-bowel fermentation of resistant starch increases butyrate production [25]. Consequently, oral administration of resistant starch could benefit IBD patients by increasing butyrate levels in their gut.

A dietary supplement containing CS and prebiotics (resistant starch, β-glucans and mannanoligosaccharides (MOS)) was developed to target intestinal inflammation, oxidative stress and gut dysbiosis. Our research hypothesis was that long-term administration of this supplement is safe and helps to decrease intestinal inflammation and oxidative stress and restore normobiosis. The purpose of this study was therefore to evaluate the effects of this supplement on clinical disease activity, intestinal histology, gut microbiota, and selected serum biomarkers in dogs with IBD over a time course of 180 days.

## Methods

### Dogs

This was a multicentric randomized double-blind placebo-controlled study using client-owned dogs. All animal owners gave written informed consent. The protocol was reviewed and approved by the Animal Experimentation Committee of the Complutense University of Madrid (UCM). Both the owners and the investigators were blinded to treatment assignment until the end of the study.

Inclusion criteria included dogs with a minimum age of 1 year, persistent (>3 weeks in duration) gastrointestinal signs, confirmation of IBD by histologic evaluation of biopsy samples and a baseline score of at least 4 on the canine IBD activity index (CIBDAI) [1]. A diagnostic exclusion protocol was carried out in all patients in order to rule out other possible causes of chronic diarrhea, including urinalysis, abdominal ultrasound, fecal exam, CBC and serum biochemistry. Animals were excluded if they presented with significant extra-intestinal disease, intestinal neoplasia, or severe hypoproteinemia (serum albumin <1.8 g/dL), if they had not been adequately dewormed within 60 days prior to trial entry, and if they had received antibiotics, prebiotics or probiotics 7 days prior to inclusion, or glucocorticoids 14 days prior to inclusion. Pregnant bitches were excluded.

Treatment was initiated 15 days after endoscopy. Dogs with biopsy-confirmed IBD were then randomized in a 1:1 allocation ratio by means of a computer-generated schedule into one of the 2 treatment groups: placebo and supplement. All dogs were switched to a hydrolyzed diet (Hypoallergenic, Royal Canin Ibérica SA, Madrid, Spain) which was maintained during the course of the study. Dogs in the placebo group (group A; *n* = 14) received a placebo powder (containing only excipients and flavorings) orally once daily, and dogs in the supplement^1^ group (group B; *n* = 13) received an oral daily dose per kg of bodyweight of 215 mg α-glucan butyrogenic resistant starch, 10 mg CS, and 26 mg β-glucans and MOS for 180 days. A rescue protocol was available in the case of clinical relapse; specifically, if the veterinarian responsible for the case decided that a considerable worsening had occurred since last visit, patients were administered a combination of metronidazole (10 mg/kg, PO, BID) for 21 days, ranitidine (5 mg/kg, PO, SID) and metoclopramide (0.5 mg/kg, PO, BID) for 60 days, and tapering doses of oral prednisone (1 mg/kg BID for 10 days, followed by 0.5 mg/kg BID for 10 days, 0.5 mg/kg SID for 10 days, and 0.5 mg/kg EOD for 30 days) [26]. Relapsed cases that received the rescue protocol were excluded from the final data analysis.

### Evaluations

CIBDAI scores and serum biomarkers were monitored at the initial visit (15 days prior to treatment start) and after 30, 60, 90, 120 and 180 days of treatment. Fasting blood samples were collected to measure serum biomarkers of inflammation (C-reactive protein (CRP)) and oxidative stress (PON1 and total antioxidant capacity (TAC)). In addition, serum albumin and cholesterol were measured. The decrease in these two analytes in canine IBD has been associated with a worse histological score, because they are lost due to damaged intestinal villi and lacteals [27]. Albumin concentrations are also related to the severity of IBD in humans [28], and in dogs [10]. In human IBD patients, cholesterol levels show a negative correlation with disease activity [29]. CRP concentrations were measured using an immunoturbidimetric assay (CRP OSR 6147 Olympus Life and Material Science Europe GmbH, Hamburg, Germany) [30]. PON1 activity was determined using *p*-nitrophenyl acetate as substrate [31]. Serum TAC was measured by the method proposed by Erel et al. [32] with a minimum detection limit of 0.01 mmol/L. Total cholesterol and albumin analyses were performed using commercially available kits. All assays were performed in an automated clinical chemistry analyzer (Olympus AU600, Olympus Diagnostica GmbH, Freiburg, Germany).

To assess changes in gut microbiota, fecal samples were analyzed by PCR-restriction fragment length polymorphism (PCR-RFLP). Samples were collected at the initial visit and 120 days after treatment was initiated. All analyses were performed by a single investigator (MCC). Immediately after collection, 1 g of fecal sample was placed in 3 mL of 98 % grade ethanol and cool transported to CReSA lab (campus Autonomous University of Barcelona (UAB, Barcelona, Spain)) where it was kept at 4 °C until DNA extraction, as described elsewhere [33, 34]. A sample of 400 mg from the ethanol-preserved feces was washed twice with sterile buffered peptone water. DNA was extracted using the QIAamp DNA Stool Minikit (Qiagen Inc., Chatsworth, CA) following the manufacturer’s instructions with minor modifications including a lysozyme incubation step and adding Ribonuclease-A and BSA (Sigma Chemical Co., St. Louis, MO, USA) to the eluted DNA [35]. The 16S rDNA was amplified as previously described [35]. Aliquots of the amplified DNA fragments were digested in separate tubes with 5 restriction endonucleases (Alu I, Rsa I, Hpa II, Sau 3A I, Cfo I; F. Hoffmann-LaRoche Ltd. Group, Basel, Switzerland). The endonuclease fragments were resolved in 2 % agarose gels at 150 V for 60 min. DNA bands were visualized using an imaging system (UV Chemigenious Image System; SynGene, Cambridge, UK) and the GeneSnap software (SynGene, Frederick, MD). Dendrograms showing the percentage of similarity among PCR-RFLP band patterns were generated on the basis of the Manhattan distance [36].

In order to evaluate histologic changes, upper and lower gastrointestinal endoscopies were performed at the initial visit and after 180 days of treatment. Multiple biopsy samples from stomach (4–8 samples, considered adequate by the pathologist (ARB)), small intestine (8–18 samples) and large intestine (8–18 samples) were obtained using different flexible video endoscopes depending on the size of the patient (Fujinon® EG-200FP, EG-270NS and EC-200LR, Fujifilm Holdings Corporation, Tokyo, Japan) and sent to Citopath Pathology Service (private laboratory) (UCM) for microscopic review. Samples were fixed in formalin, routinely processed, and embedded in paraffin. Serial sections from paraffin tissue blocks were cut for routine hematoxylin and eosin staining. Microscopic examination (Leica DM2000 LED Professional Microscope with MC170 HD Digital Camera, Leica Biosystems, Barcelona, Spain) of all tissues was performed by a single pathologist (ARB) who was blinded to treatments, and objectively graded endoscopic specimens following the WSAVA (World Small Animal Veterinary Association) histopathologic Guidelines for the Diagnosis of Idiopathic Inflammatory Bowel Diseases in Dogs and Cats [37]. Histologic scores for stomach, duodenum and colon were also evaluated and compared between groups.

Concomitant treatments were recorded for all animals during the study, and relapse rates were compared between groups.

### Statistical analysis

Statistical analysis was performed by a biostatistician (SMS) with the use of statistical software.^2^ A one-way analysis of variance (ANOVA) of repeated measures and an uncorrected Fisher’s Least Significant Difference (LSD) test were used to compare the values obtained at different times during treatment with the pretreatment values. Unpaired *t*-test was performed to compare values between groups at each visit. Fisher’s exact test was performed to analyze differences in relapse rates between groups. Analytes that did not follow a parametric distribution were log-transformed before applying the test.

## Results

**Fig. 1:**
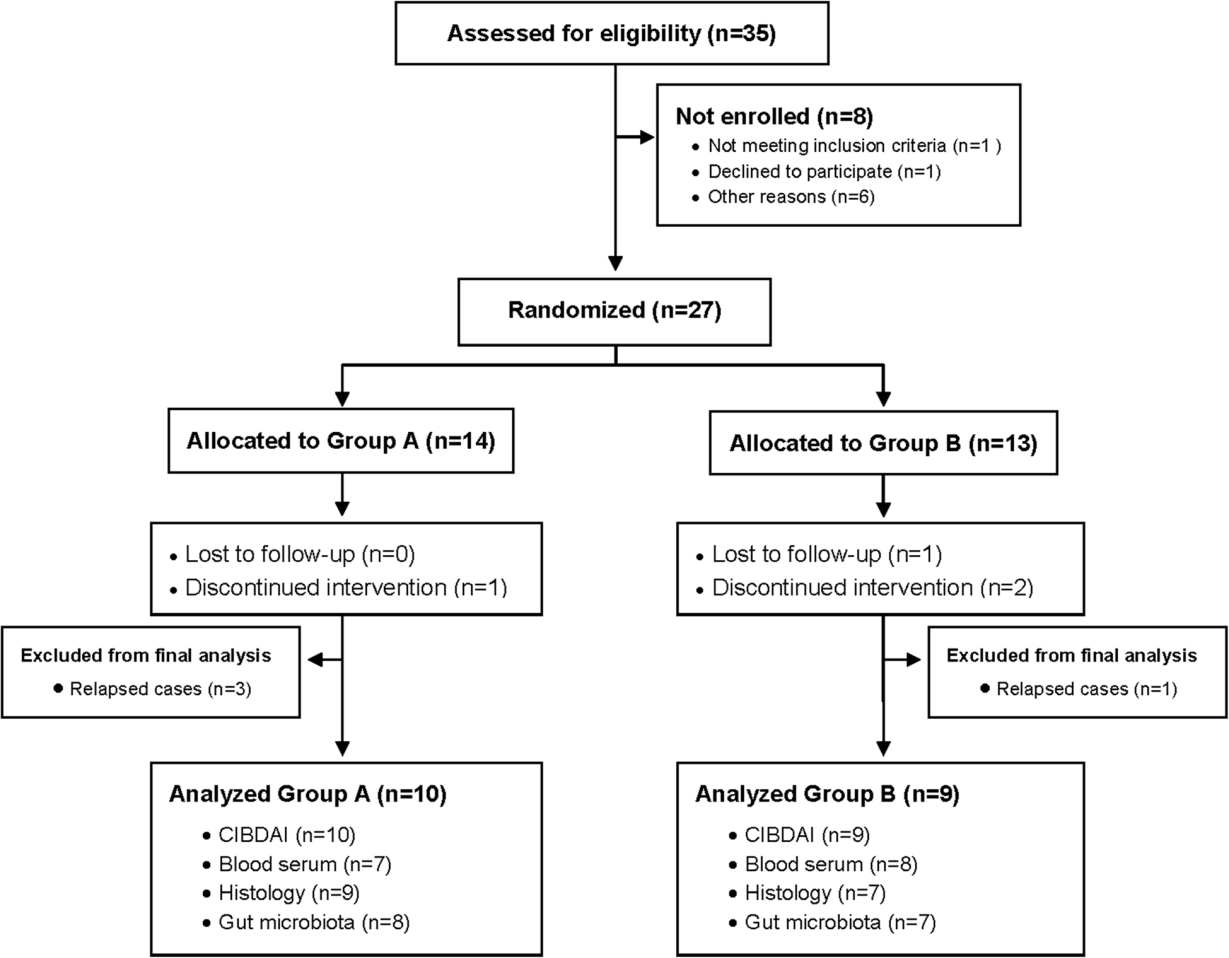
Flow diagram of IBD dogs. *Group A*, placebo; *group B*, supplement

During the study period, 35 dogs were assessed for eligibility (33 recruited by CPV Raspeig, Alicante; 1 by CV Mediterráneo, Madrid; and 1 by HV Tomás Bustamante, Cantabria) and 27 of them were randomized into the clinical trial (Fig. 1). Of the 27 dogs enrolled, 14 were randomized to Group A (placebo) and 13 to Group B (supplement). Breeds included Yorkshire terrier (6), crossbreed (5), poodle (2), French bulldog (2), Samoyed (2), German shepherd (2), and 1 dog each of the following breeds: Basset hound, Lhasa Apso, Great Dane, Siberian Husky, Shar-Pei, Chihuahua, Beagle, and miniature schnauzer; 19 of the dogs were males and 8 were females, ranging in age from 1 to 15 years (mean 4.85 ± 3.63 years). A total of four dogs (14.8 %) did not complete the study. In Group A, one dog discontinued the intervention due to owner decision; in Group B, 2 dogs were excluded due to owner decision (non-compliance) and 1 dog was lost to follow-up. Overall, 13 dogs in Group A (9 with second endoscopy) and 10 dogs in Group B (7 with second endoscopy) completed the full clinical trial.

### Canine IBD activity index

**Fig. 2:**
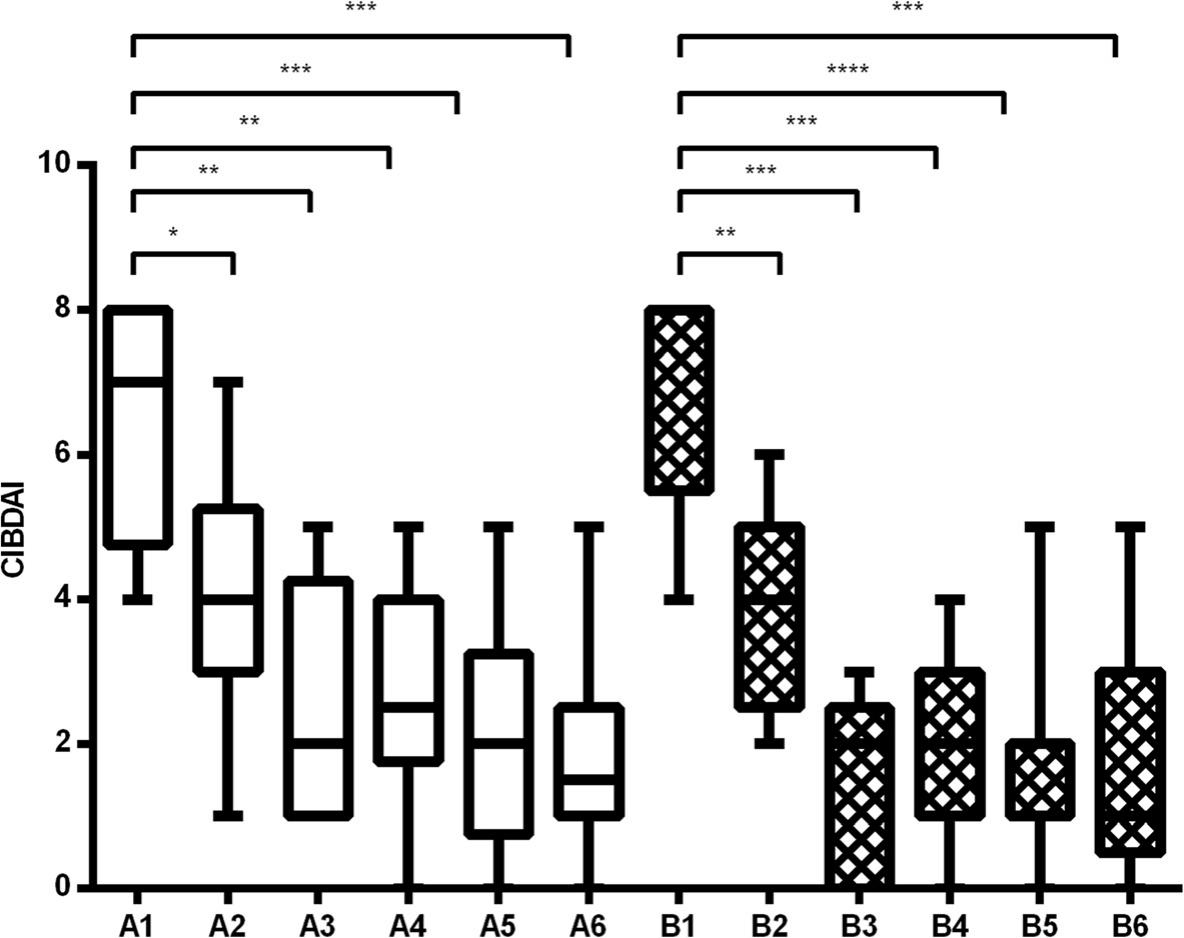
One-way ANOVA CIBDAI scores in group *A* (placebo) and *B* (supplement) 15 days before treatment start (*1*), and at 30 (*2*), 60 (*3*), 90 (*4*), 120 (*5*) and 180 (*6*) days of treatment. *Asterisks* indicate statistical significance compared to pretreatment values (*p < 0.05; **p < 0.01; ***p < 0.001; ****p < 0.0001)

Figure 2 shows changes in median CIBDAI scores. At the time of diagnosis, there were no significant differences (*p* = 0.62) between groups (median (25th–75th percentile): placebo = 7 (4.75–8), supplement = 8 (5.5–8)). After treatment, a significant reduction in median CIBDAI scores was observed in both groups (*p* < 0.001). Compared to baseline median CIBDAI scores, the supplement group showed a 2-fold decrease (*p* < 0.01) and a 4-fold decrease (*p* < 0.001) after 1 and 60 days of treatment, respectively. In the placebo group, decreases in median CIBDAI scores after 30 and 60 days of treatment were 1.75-fold (*p* < 0.05) and 3.5-fold (*p* < 0.01), respectively. No significant differences between groups were found at any time point.

### Serum biomarkers

Initially, there were no statistically significant differences (*p* > 0.05) between groups for any of the serum biomarkers analyzed. Initial median (25th–75th percentile) values were within the reference ranges of our laboratory, with the exception of a decreased PON1 in all dogs (placebo = 1.7 (1.58–2.21); supplement = 2.02 (1.88–2.57); reference values = 3–4.3 IU/mL), increased CRP in the placebo group (13.5 (5.0–17.8); reference values <12 μg/mL), and decreased TAC in the supplement group (0.25 (0.16–0.36); reference values >0.35 mM/L).

**Fig. 3:**
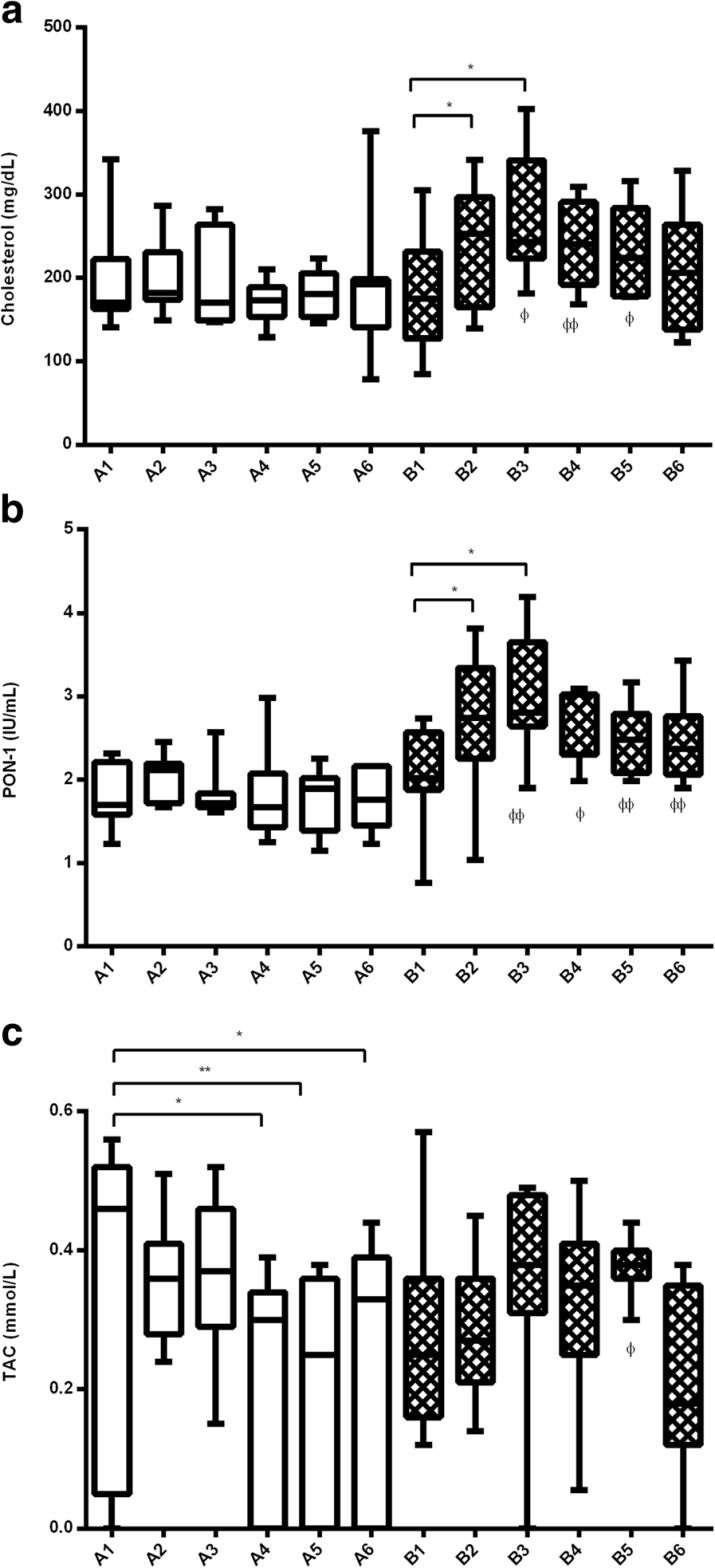
Concentrations of serum cholesterol (**a**), PON1 (**b**) and TAC (**c**) in groups *A* (placebo) and *B* (supplement) at the initial visit (*1*; 15 days before treatment start), and at 30 (*2*), 60 (*3*), 90 (*4*), 120 (*5*) and 180 (*6*) days of treatment. *Asterisks* indicate statistical significance compared to pretreatment values within each group (one-way ANOVA; *p < 0.05; **p < 0.01). Differences between groups are also represented (unpaired *t*-test; ϕ*p* < 0.05; ϕϕ*p* < 0.01)

Compared with pretreatment values, the supplement group showed significantly increased cholesterol and PON1 after 30 and 60 days of treatment (*p* < 0.05) and a significantly decreased TAC was observed in the placebo group from 90 days of treatment (*p* < 0.05). When groups were compared, the supplement group showed significantly higher cholesterol after 60 (*p* < 0.05), 90 (*p* < 0.01) and 120 (*p* < 0.01) days of treatment, and significantly higher (*p* < 0.01) PON1 after 60, 90, 120 and 180 days of treatment compared to placebo. During the study, in each group one dog showed cholesterol values above the reference range (120–300 mg/dL) and one below it. PON1 remained below the reference range (3–4.3 IU/mL) in all dogs in the placebo group. The placebo group showed significantly lower TAC levels after 120 days of treatment (*p* < 0.05) (Fig. 3). All dogs in the study showed TAC values below the reference range (>0.35 mM/L) at some time point, but only with the supplement all dogs finished the study with levels within it. No significant changes were seen for the other serum biomarkers.

### Histologic examinations

**Fig. 4:**
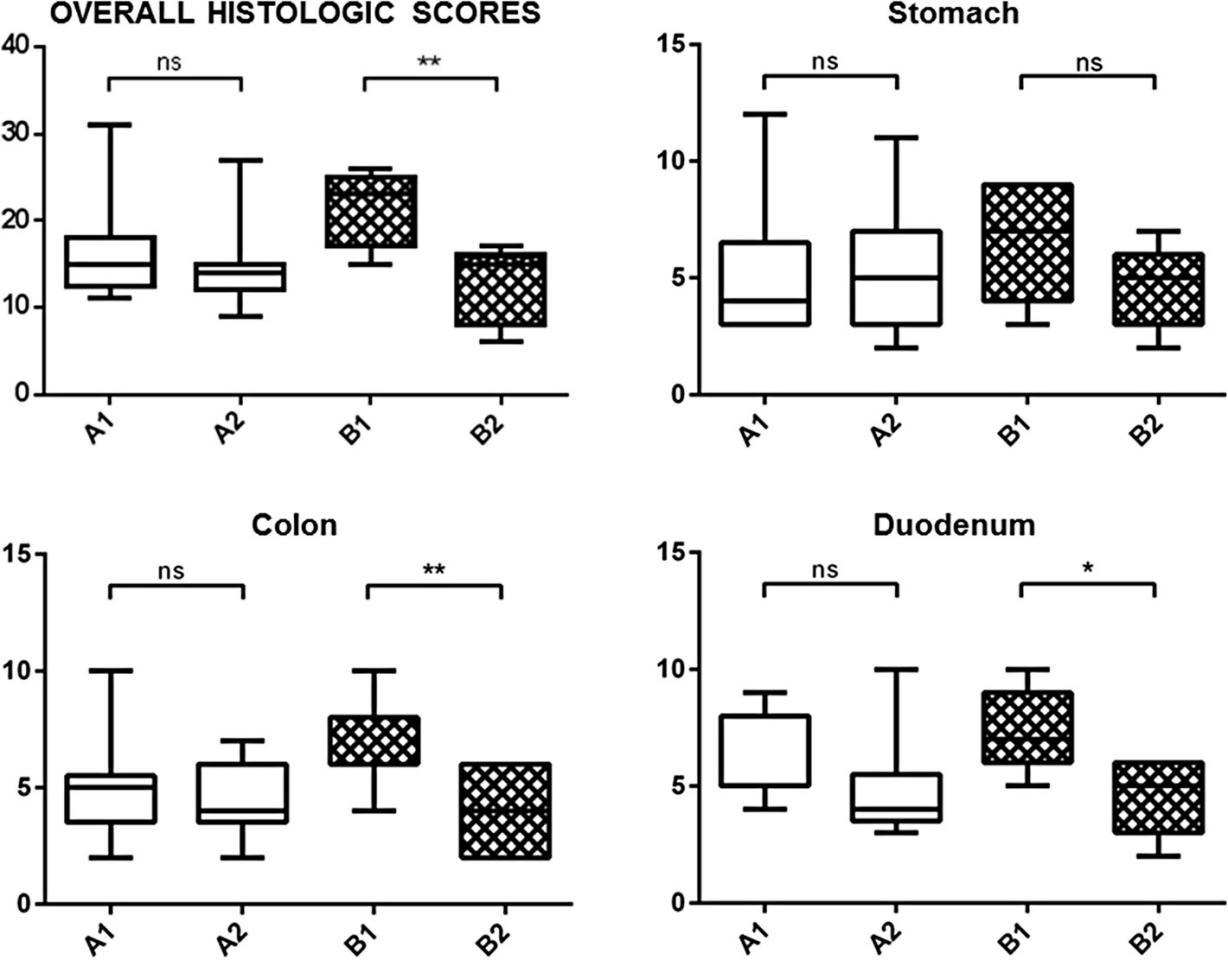
WSAVA histologic scores (overall and for each specific tissue–stomach, colon and duodenum) in groups *A* (placebo) and *B* (supplement) at initial visit (*1*) and after 180 days of treatment (*2*; 195 days after initial visit) treatment. *Asterisks* indicate statistical significance compared to pretreatment (*t* = 0 days) values (one-way ANOVA; *p < 0.05; **p < 0.01; *ns* not statistically significant)

Variations in histologic scores are shown in Fig. 4. At the time of diagnosis, there were no significant differences between groups in overall WSAVA histologic scores (median (25th–75th percentile) group A = 15 (12.5–18); group B = 23 (17–25); *p* = 0.09). A second biopsy was not possible in 3 of the 19 patients that finished the study without needing the rescue protocol (placebo: *n* = 1; supplement: *n* = 2) due to reluctance of some owners to put their pet through another anesthetic procedure. After treatment, the supplement group showed a significant 1.53-fold decrease (*p* < 0.01) in median overall histologic score, whereas a non-significant 1.07-fold decrease (*p* = 0.63) was observed in the placebo group. With the supplement, a significant decrease was seen in duodenum (*p* = 0.02) and colon (*p* = 0.0013). No statistically significant differences were found with the placebo for any tissue. When groups were compared, there were no significant differences at any time point.

### Gut microbiota

**Fig. 5:**
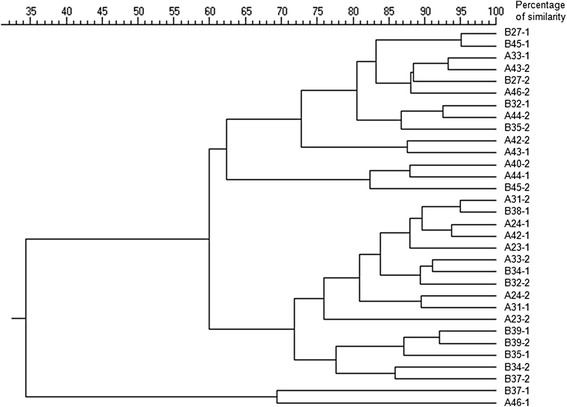
Ecological changes in microbial populations measured by PCR-RFLP from fecal samples from groups *A* (placebo) and *B* (supplement) at initial visit (*1*) and at the end of the treatment (*2*; 135 days after first measurement). The dendrogram illustrates the percentage of similarity among DNA band patterns

The dendrogram formed with the band patterns generated by PCR-RFLP of the fecal samples is shown in Fig. 5. No bands were generated in the PCR-RFLP electrophoresis gel from samples belonging to two animals from different groups, probably due to an insufficient amount of DNA. Consequently, these two study subjects were excluded from the dendrogram. All but two samples were grouped in two main clusters at similarity levels of 62 and 72 %, respectively. Before and after treatment, samples from both the placebo and the supplement groups were not grouped together, but scattered throughout the dendrogram instead. Thus, no differences were observed between the placebo and the supplement groups or between initial and final samples of either group.

### Clinical relapses and side effects

During the study, clinical relapses were observed in 3/14 dogs in the placebo group (21.4 %) and in 1/13 dogs in the supplement group (7.7 %). No statistically significant differences were found in the proportion of animals that relapsed in each group (*p* = 0.59). In the placebo group, two dogs relapsed at 60 and 90 days of treatment and received the rescue protocol. The third dog in the placebo group relapsed after 30 days of treatment, and received oral prednisone 1 mg/kg SID for 10 days. In the supplement group, one case relapsed after 90 days of treatment and started the rescue treatment. Overall, both treatments were well tolerated and no side effects were reported in any of the groups.

## Discussion

The etiology of canine IBD is not fully understood, but there are some factors that are thought to be implicated in causing intestinal inflammation including an abnormal immune response, genetic susceptibility, nutrition and the intestinal microbiota [3–5]. Herein, we report preliminary data on the efficacy and safety of a dietary supplement containing CS and prebiotics in dogs with IBD, which might suggest its usefulness to further extend clinical remission periods and avoid excessive usage of glucocorticoids and antibiotics, hence minimizing their unwanted side effects.

A significant decrease in CIBDAI was observed in both groups at the end of the study. This clinical improvement regardless of the treatments received may be explained by a positive response to the hydrolyzed diet alone [4, 9].

To the authors’ knowledge, this is the first study to report measurements of PON1 in dogs with biopsy-confirmed IBD. Initially, serum values below our laboratory reference range for PON1 were found in all study dogs, which is in line with what has been observed in human patients with IBD [16]. After treatment, an increased PON1 was observed only in the supplement group. This could reflect a protective effect of the supplement against higher oxidative stress occurring in IBD. In addition, in our study, serum cholesterol increased only in the supplement group. This could indicate an improvement in the IBD since a decrease in serum cholesterol has been related to a worse histological score in dogs with IBD, probably because damaged intestinal villi and lacteals produce cholesterol loss [27]. In human IBD patients, cholesterol levels show a negative correlation with disease activity and inflammatory indices [29]. No significant changes in CRP were observed after treatment in this study. Although increased concentrations of CRP have been correlated with clinical disease activity in human patients with IBD and this marker is considered adequate for evaluating response to treatment in canine IBD [1, 38], other studies found that CRP was not a good indicator of disease severity in dogs [39]. We found significantly decreased TAC values after treatment only with the placebo, which could indicate a higher susceptibility to oxidative tissue damage compared to the supplement group. The differences found in our study between PON1 and TAC might be explained by the fact that TAC measures all the antioxidants of a sample, since it represents the measurement of the amount (in moles) of a free radical scavenged by a test solution [40, 41], and includes many antioxidants in addition to PON1. Therefore, depending on the behaviour of the different individual antioxidants, differences might appear between values of PON1 (which is just one component) and TAC (which is the sum of all antioxidants). In addition, PON1 is also affected by inflammation in dogs [15], which could also contribute to its different behaviour. Overall, serum results from this study could suggest a reduction of oxidative stress in the supplement group.

Compared to baseline histologic scores, a significant improvement was seen only in the supplement group. Furthermore, these significant improvements were limited to the small and large intestines (especially the colon), which corresponds with the target tissues of the supplement. Nevertheless, the difference between groups in median WSAVA histologic score after treatment did not reach the level of statistical significance.

In our study, a clear change in microbial population was not seen in both groups despite the administration of prebiotics. This could be attributable to the heterogeneity of our study subjects, which belonged to different breeds, age groups, and geographical regions with different environmental conditions. It could also be related to the small sample size of the study. Moreover, this concurs with the findings of another recent study describing clinical improvements in dogs with IBD after medical therapy which were not accompanied by significant changes in their fecal microbiome [5].

Clinical relapses were seen earlier and more frequently in dogs belonging to the placebo group, although differences between groups were not statistically significant probably due to the study being underpowered.

Although the results of the present study provide useful information about the effects of CS and prebiotics in dogs with IBD, there are some limitations that should be mentioned. First, the sample size was small and this might have affected the level of statistical significance of certain parameters. Due to this concern, although better serological results were obtained with the supplement indicating and improved intestinal absorption capacity and decreased inflammation and oxidative stress, the observed improvements in histopathology and CIBDAI could not be statistically supported. It should also be mentioned that male dogs and Yorkshire terriers were overrepresented in this study, and that most cases came from one veterinary center (CPV Raspeig). Lastly, PCR-RFLP was selected as the technique for evaluating changes in gut microbiota. Ideally, other methods could have been used, but the authors chose PCR-RFLP based on prior publications reporting its usefulness [35, 42].

## Conclusions

Our results suggest a beneficial effect of long-term oral administration of a dietary supplement containing CS and prebiotics combined with a hydrolyzed diet in dogs with IBD by increasing serum PON1, TAC and cholesterol in dogs with IBD. The administration of the supplement over 180 days was safe. A supplemental effect to dietary treatment was not statistically demonstrated for CIBDAI, histology and relapse rates, likely because the study was underpowered. Further studies with larger randomized clinical trials are warranted to confirm these results.

## Abbreviations

IBD: inflammatory bowel disease
CS: chondroitin sulfate
NF-κB: nuclear factor kappa-light-chain-enhancer of activated B cells
PON1: paraoxonase-1
SCFA: short chain fatty acids
MOS: mannanoligosaccharides
UCM: Complutense University of Madrid
CIBDAI: canine IBD activity index
CRP: C-reactive protein
TAC: total antioxidant capacity
PCR-RFLP: PCR-restriction fragment length polymorphism
WSAVA: World Small Animal Veterinary Association
ANOVA: analysis of variance
LSD: least significant difference

## Footnotes

1 Entero-Chronic®, Bioiberica SA, Barcelona, Spain.

2 GraphPad Prism 6, GraphPad software, Inc, San Diego, USA.
